# Association of ferritin levels with depression, anxiety, sleep quality, and physical functioning in patients with fibromyalgia syndrome: a cross-sectional study

**DOI:** 10.3325/cmj.2019.60.515

**Published:** 2019-12

**Authors:** Sevil Okan, Ayla Çağlıyan Türk, Hakan Şıvgın, Filiz Özsoy, Fatih Okan

**Affiliations:** 1 Department of Physical Medicine and Rehabilitation, Tokat State Hospital, Tokat, Turkey; 2 Department of Physical Medicine and Rehabilitation, Hitit University Faculty of Medicine, Çorum, Turkey; 3 Department of Internal Medicine, Tokat State Hospital, Tokat, Turkey; 4 Department of Psychiatry, Tokat State Hospital, Tokat, Turkey; 5 Department of Public Health Nursing, Tokat Gaziosmanpaşa University Faculty of Health Science, Tokat, Turkey

## Abstract

**Aim:**

To determine the frequency of ferritin deficiency in individuals with fibromyalgia syndrome (FMS) and to evaluate the association of ferritin level with depression, anxiety, sleep quality, and physical functioning.

**Methods:**

This cross-sectional study, conducted from 2016 to 2017, compared the frequency of ferritin deficiency between 100 non-anemic fibromyalgia patients and 100 non-anemic individuals without FMS. Serum ferritin level of <30 ng/mL indicated iron deficiency. FMS patients filled out demographic questionnaire, Fibromyalgia Impact Questionnaire, Beck Anxiety Inventory, Beck Depression Inventory, and Pittsburg Sleep Quality Index.

**Results:**

Median serum ferritin level was 20.95 ng/mL. A total of 64% of patients and 42% of controls had iron deficiency. Beck Anxiety Inventory, Beck Depression Inventory, and Pittsburgh Sleep Quality Index scores were not associated with ferritin levels. FMS patients with poor sleep quality had significantly higher Beck Depression Inventory, Beck Anxiety Inventory, and Fibromyalgia Impact Questionnaire scores (*P* < 0.05). In individuals with poor sleep quality, lower ferritin levels also correlated with higher Beck Depression Inventory scores (r = -0.277, *P* < 0.05). Sleep quality was not significantly associated with age, body mass index, duration of diagnosis, and serum ferritin levels.

**Conclusions:**

Patients with fibromyalgia syndrome have a rather high prevalence of non-anemic iron deficiency. No associations were found between serum ferritin level and anxiety, depression, sleep quality, and physical functioning.

ClinicalTrials.gov identifier: NCT03825393.

Fibromyalgia syndrome (FMS) is a musculoskeletal disease characterized by chronic pain, fatigue, anxiety, memory loss, and morning stiffness (1). FMS prevalence in the general population is 2.7% and is three times greater in women (2). While the risk factors for the disease include vitamin and mineral deficiencies, its exact etiology and pathogenesis are unclear. The onset and prognosis of FMS are determined by interactions between neuroendocrine, immunological, and metabolic factors, and the diagnosis is usually established in middle-aged patients (2,3).

Serum ferritin concentration (cut-off <30 ng/mL) is the most sensitive and specific test for iron deficiency (4-6). The symptoms specific to a decreased activity of iron-containing enzymes are weakness, fatigue, lack of concentration, lower work performance, and impaired oxygen transport to body tissues. However, it is not known to what extent these non-hematological effects of iron deficiency emerge before anemia occurs (7). Iron deficiency in FMS causes chronic fatigue, myalgia, decreased endurance, and sleep disorders (8,9). It lowers the pain threshold, making increased pain sensitivity a potential key factor in the pathophysiology of fibromyalgia (10). A case-control study by Ortancil et al (11) showed FMS patients had lower serum ferritin levels than healthy control groups. To the best of our knowledge, no study has so far evaluated the association between ferritin levels and sleep in FMS patients. Therefore, the aim of the present study was to determine the frequency of non-anemic ferritin deficiency in individuals with FMS and to evaluate the association of ferritin levels with anxiety, depression, sleep quality, and physical functioning, as well as the interactions between clinical parameters in FMS patients.

## PARTICIPANTS AND METHODS

This cross-sectional study included women older than 18 who presented to Physical Medicine and Rehabilitation polyclinic and whose ferritin levels had been measured in the previous four weeks at the Tokat State Hospital in the 2016-2017 period. Exclusion criteria were parenteral or enteral iron use in the last four weeks, hemoglobin level less than 12 g/dL, infection, malignity, active inflammatory arthritis, serious skin variations, serious peripheral vascular disease, iron storing disorder, and pregnancy or lactation. After the exclusion criteria were applied, 100 participants in the patient group were selected among 137 non-anemic FMS patients who were diagnosed based on 2011 FMS diagnostic criteria of the American Rheumatology College (12). The selection was performed by simple random sampling with random number generator. The control group included 100 women selected by a simple random sampling among 153 non-anemic women without FMS. The study was approved by the Ethics Committee for Clinical Research of Gaziosmanpasa University (17-KAEK-093), and all participants gave informed consent. Serum ferritin levels were obtained from hospital information system records. Only FMS patients filled out a demographic questionnaire, Beck Anxiety Inventory (BAI), Beck Depression Inventory (BDI), Fibromyalgia Impact Questionnaire (FIQ), and Pittsburgh Sleep Quality Index (PSQI).

FIQ is a 10-item evaluation tool measuring the status, prognosis, and outcomes of FMS patients. The total score ranges from 0 to 100, with a higher score indicating a greater effect of FMS on functionality. The validity and reliability of FIQ in Turkish population were assessed by Sarmer et al  (13).

BDI is a 21-item questionnaire evaluating the presence and severity of depression. A higher score indicates more severe depression (0-9 points: minimum depression; 10-18 points: slight depression; 19-29 points: moderate depression; 30-63 points: severe depression). The validity and reliability of BDI in Turkish population were assessed by Hisli et al (14).

BAI is a short, 21-item questionnaire evaluating the severity of anxiety. The total score ranges from 0 to 63 (0-9 points: normal; 10-18 points: slight to moderate anxiety; 19-29 points: moderate to severe anxiety; and 30-63 points: very severe anxiety). The validity and reliability of BAI in Turkish population were assessed by Ulusoy et al (15).

PSQI is an 18-item questionnaire measuring sleep quality and disorders in a period of one month. PSQI measures subjective sleep quality, duration and latency of sleep, sleep disorders, habitual sleep efficiency, daytime dysfunction, and sleep medications use. A score of 5 or higher indicates poor sleep, while a score lower than 5 indicates good sleep quality. The validity and reliability of PSQI in Turkish population was assessed by Ağargün et al (16).

Serum ferritin level was measured with Roche Cobas e801 autoanalyzer (Roche Diagnostics, Mannheim, Germany) in our hospital laboratory with use of electrochemiluminescence immunoassay method. Serum ferritin level lower than 30 ng/mL indicated iron deficiency.

### Statistical analysis

General characteristics of study groups were summarized by use of descriptive statistics. Normality of distribution was assessed with the Shapiro-Wilk’s test. Continuous variables are expressed as mean ± standard deviation for normally distributed variables and median and range for non-normally distibuted variables, while categorical variables are expressed as counts and percentages. Group means were compared with use of the independent-samples *t* test or Mann-Whitney U test, where appropriate. Correlations between quantitative variables were evaluated with Pearson correlation coefficient. The level of statistical significance was set at *P* < 0.05. Statistical analyses were carried out using SPSS software, version 19.0. (IBM Corp, Armonk, NY, USA).

## RESULTS

Control and patient groups did not significantly differ in mean age (42.24 ± 8.03 and 40.44 ± 8.47 years, respectively). Average body mass index (BMI) in the patient group was 29.56 ± 5.68 kg/m^2^. Average duration of diagnosis was 21.5 months in 73% of patients, while 27% of patients were diagnosed during their visit to our outpatient clinic (Table 1). Significantly more patients than controls had serum ferritin level lower than 30 ng/mL (64 vs 42, *P* = 0.002). Control group had a significantly higher ferritin level (median 34.91, IQR 17.87-55.12 ng/mL] than FMS patients (median 20.95, IQR 11.75-39.55 ng/mL, *P* < 0.001).

**Table 1:** Quantitative variables in individuals with fibromyalgia syndrome

	Mean ± standard deviation	Minimum	Maximum
Age (years)	42.24 ± 8.03	21.00	59.00
Height (cm)	1.61 ± 0.08	1.15	1.82
Weight (kg)	75.7 ± 12.2	40.00	117.00
Body mass index (kg/m^2^)	29.56 ± 5.68	15.62	52.93
Duration of diagnosis in months (median and IQR*)	0 (0-1.25)	0.00	120.00
Ferritin (ng/mL, median and IQR)	20.95 (11.75-39.55)	2.75	93.00
Fibromyalgia Impact Questionnaire	61.69 ± 17.31	14.63	95.63
Beck Depression Inventory	15.73 ± 9.76	0.00	45.00
Beck Anxiety Inventory	22.58 ± 11.14	0.00	54.00
Pittsburgh Sleep Quality Index	8.3 ± 3.77	1.00	18.00

*IQR – interquartile range.

FMS patients were further divided into iron-deficiency and non-deficiency group. The groups did not differ in BMI, duration of diagnosis, FIQ, BDI, BAI, and PSQI scores, although iron deficiency group had significantly lower average age (*P* = 0.047). It also had relatively higher BDI, BAI, and PSQI scores, but the differences were not significant (Table 2).

**Table 2:** Distribution of quantitative variables according to iron deficiency (<30 ng/mL) in patients with fibromyalgia syndrome

Variables*	Patients with ferritin level		*P*^†^
	<30 ng/mL (n = 64)	≥30 ng/mL (n = 36)	
Age (years)	41.05 ± 7.69	44.36 ± 8.28	0.047
Height (cm)	1.61 ± 0.09	1.60 ± 0.07	0.709
Weight (kg)	75.08 ± 12.03	76.81 ± 12.59	0.500
Body mass index (kg/m^2^)	29.31 ± 6.00	30.01 ± 5.11	0.560
Duration of diagnosis (months)	0 (0-1.75)	0 (0-1.25)	0.818^‡^
Fibromyalgia Impact Questionnaire	61.52 ± 17.4	62.00 ± 17.39	0.895
Beck Depression Inventory	17.03 ± 10.66	13.42 ± 7.51	0.075
Beck Anxiety Inventory	23.34 ± 11.55	21.22 ± 10.40	0.363
Pittsburgh Sleep Quality Index	8.36 ± 3.81	8.19 ± 3.75	0.835

*Data are presented as mean ± standard deviation or median and interquartile range.

†Independent samples *t* test.

‡Mann-Whitney U test.

According to the PSQI score, FMS patients were divided into poor sleep and good sleep quality group. Poor sleep quality group had significantly higher BDI, BAI, and FIQ scores (*P* < 0.05) (Table 3). Sleep quality was poor in 76.57% (n = 49) of individuals with iron deficiency and in 77.7% (n = 28) of individuals with no deficiency (*P* = 0.999).

**Table 3:** Distribution of quantitative variables based on sleep quality in patients with fibromyalgia syndrome

Variables*	Patients with sleep quality		*P*^†^
	poor (n = 77)	good (n = 23)	
Age (years)	43.00 ± 7.91	39.70 ± 8.06	0.083
Height (cm)	1.61 ± 0.08	1.59 ± 0.08	0.408
Weight (kg)	76.99 ± 11.96	71.39 ± 12.27	0.053
Body mass index (kg/m^2^)	29.96 ± 5.80	28.23 ± 5.16	0.203
Duration of diagnosis (months)	0 (0-1)	0 (0-2.5)	0.912^‡^
Fibromyalgia Impact Questionnaire	28.32 ± 21.14	25.52 ± 21.72	0.580
Beck Depression Inventory	65.99 ± 15.36	47.31 ± 15.90	<0.001
Beck Anxiety Inventory	16.78 ± 9.87	12.22 ± 8.71	0.049
Pittsburgh Sleep Quality Index	24.78 ± 10.83	15.22 ± 8.94	<0.001

*Data are presented as mean ± standard deviation or median and interquartile range.

†Independent samples *t* test.

‡Mann-Whitney U test.

A significant correlation between BDI and PSQI (r = 0.368, *P* < 0.05) was observed in participants with iron deficiency but not in those with no deficiency. In poor sleep quality group, BDI scores increased significantly with decreasing ferritin levels (r = -0.277, *P* < 0.05).

## DISCUSSION

The present study showed that iron deficiency was significantly more frequent in FMS patients than in controls. Similarly, Ortancil et al (11) found the FMS incidence risk to be 5.9 times higher in patients with ferritin levels lower than 50 ng/mL, suggesting an association of a relative decrease in iron reserve with FMS (11). Another study found a high FMS prevalence in patients with iron deficiency anemia (17). In addition, FMS patients in our study had lower median ferritin level than the control group. Similarly, another study found that women with FMS compared with controls also had lower calcium, magnesium, iron, and manganese hair concentrations (18). Contrary to these findings, in a study by Mader et al (19) FMS patients did not have lower serum iron, and low ferritin levels were not associated with FMS.

Mice fed on iron-deficient diet (10) had a decreased pain threshold, which was accompanied by elevated c-Fos expression in immunoreactive cells in the ipsilateral dorsal horn, indicating that iron deficiency indirectly increases cell activity at the spinal cord level (10). In FMS, which is characterized by chronic widespread pain, IV iron supplement considerably improved pain and fatigue index (20). In this disease, iron deficiency-related changes could also be observed in response to pain at the central nervous system level.

FMS patients with iron deficiency in the present study had higher depression and anxiety levels and poor sleep quality, although the differences were not significant. FMS patients are known to suffer from symptoms that considerably affect their life quality, such as fatigue, stiffness, susceptibility to cold, cognitive disorder, sensitivity to outside factors, sleep disorders, anxiety, and depression (21). This might be explained by the fact that these patients have lower biogenic amine metabolites, such as dopamine, norepinephrine, and serotonin, in the cerebrospinal fluid (22,23). Iron is necessary for neurotransmitter synthesis, and iron stores deficiencies decrease biogenic amine production (8,11). Impairments in serotonergic, GABAergic, dopaminergic, and other neurotransmitter and neuropeptide systems in the cerebrospinal fluid have also been shown in depression (24). Shukla et al (25) found low levels of serotonin and its metabolite, 5-hydroxyindoleacetic acid, in the brains of rats suffering from iron deficiency. Besides, increased activity and stereotypic behaviors, attributed to impairments in dopaminergic neurons were observed in rats with non-anemic iron deficiency (26). Although depression and anxiety levels in the present study were higher in individuals with iron deficiency, no association was found between iron deficiency and FIQ scores. In contrast, another study found an association between low ferritin scores and low total FIQ scores (19).

We did not observe any effect of age, BMI, duration of diagnosis, or serum ferritin level on sleep quality. While in patients with chronic obstructive lung disease sleep quality deteriorated with lower ferritin levels (27), in our patients, it deteriorated with the increase in depression. Sleeping problems may exacerbate FMS symptoms, thus increasing the risk of both depression and impaired physical and social functioning (28). In a study by Miró et al, 99% of FMS individuals had poor sleep quality, which was a significant predictor of pain, fatigue, and maladaptive social functioning (29). Sleep and pain in FMS have a two-way relationship, which could interact with depressive symptoms. Sleep disorder, difficulty falling asleep, and deterioration in sleep quality could increase pain in FMS patients (30). Sleep disorders were also associated with low ferritin levels in children with attention deficit hyperactivity disorder (31).

In the present study, individuals with poor sleep quality had higher depression when they had low ferritin levels. Another study found iron deficiency in 29.5% of individuals with depression, which was by 15% higher compared with healthy individuals (32). This finding could be explained by the fact that iron is needed for the synthesis of neurotransmitters involved in depression pathogenesis. On the other hand, Hunt et al (33) found no association between depression and ferritin levels.

Individuals with poor sleep quality in our study had poor physical functioning and higher anxiety and depression levels. In another study, sleep problems in FMS were reported to affect pain, depression, and anxiety (34). Sleep quality considerably affects health-related life quality in FMS, which is why it was proposed that sleep quality should be improved to increase the health-related life quality in these patients (35).

Despite the low number of patients in certain subgroups (BAI), this study contributed to better understanding of iron deficiency in FMS. Iron deficiency was quite common in individuals with FMS diagnosis. Also, it was reported to lower pain threshold and increase pain perception in FMS. Although no association was observed between iron deficiency and sleep quality, depression, anxiety, and physical functioning in individuals with iron deficiency, impaired sleep quality was associated with depression. In conclusion, sleep disorder, a frequently observed symptom in FMS, decreases the quality of life and increases depression and anxiety levels. Additionally, the results of our study point to the fact that FMS patients should have their ferritin levels evaluated, since iron deficiency treatment could prevent the deterioration of their clinical condition.
